# 4β-Hydroxywithanolide E Modulates Alternative Splicing of Apoptotic Genes in Human Hepatocellular Carcinoma Huh-7 Cells

**DOI:** 10.1038/s41598-017-07472-6

**Published:** 2017-08-04

**Authors:** Chien-Chin Lee, Wen-Hsin Chang, Ya-Sian Chang, Ting-Yuan Liu, Yu-Chia Chen, Yang-Chang Wu, Jan-Gowth Chang

**Affiliations:** 1 Epigenome Research Center, China Medical University Hospital, Taichung, Taiwan; 2 Department of Primary Care Medicine, Taipei Medical University Hospital, Taipei, Taiwan; 3 Department of Laboratory Medicine, China Medical University Hospital, Taichung, Taiwan; 4 Center for Precision Medicine, China Medical University Hospital, Taichung, Taiwan; 5 Graduate Institute of Natural Products, Kaohsiung Medical University, Kaohsiung, Taiwan; 6 Research Center for Natural Products and Drug Development, Kaohsiung Medical University, Kaohsiung, Taiwan; 7 Chinese Medicine Research and Development Center, China Medical University Hospital, Taichung, Taiwan; 8 Department of Bioinformatics and Medical Engineering, Asia University, Taichung, Taiwan

## Abstract

Alternative splicing is a mechanism for increasing protein diversity from a limited number of genes. Studies have demonstrated that aberrant regulation in the alternative splicing of apoptotic gene transcripts may contribute to the development of cancer. In this study, we isolated 4β-Hydroxywithanolide E (4bHWE) from the traditional herb *Physalis peruviana* and investigated its biological effect in cancer cells. The results demonstrated that 4bHWE modulates the alternative splicing of various apoptotic genes, including *HIPK3*, *SMAC*/*DIABLO*, and *SURVIVIN*. We also discovered that the levels of SRSF1 phospho-isoform were decreased and the levels of H3K36me3 were increased in 4bHWE treatment. Knockdown experiments revealed that the splicing site selection of *SMAC*/*DIABLO* could be mediated by changes in the level of H3K36me3 in 4bHWE-treated cells. Furthermore, we extended our study to apoptosis-associated molecules, and detected increased levels of poly ADP-ribose polymerase cleavage and the active form of CASPASE-3 in 4bHWE-induced apoptosis. *In vivo* experiments indicated that the treatment of tumor-bearing mice with 4bHWE resulted in a marked decrease in tumor size. This study is the first to demonstrate that 4bHWE affects alternative splicing by modulating splicing factors and histone modifications, and provides a novel view of the antitumor mechanism of 4bHWE.

## Introduction

In eukaryotic organisms, alternative splicing is mainly a co-transcriptional process that may result in a single gene coding for multiple protein isoforms with different functional and structural properties. This process can be regulated by two highly conserved protein families, namely serine-/arginine-rich (SR) proteins and heterogeneous nuclear ribonucleoproteins (hnRNPs). SR proteins often bind to splicing enhancers and activate splicing at nearby splice sites. By contrast, hnRNPs often bind to splicing silencers and exhibit the opposite activity^1–3^. For example, TRA2B antagonizes the inhibitory action of hnRNP A1 during the regulation of the alternative splicing of *SURVIVAL MOTOR NEURON* transcripts^4^, and SRSF1 antagonizes the activity of hnRNP A1 during splicing site selection^5^. This indicates that SR proteins and hnRNPs have balancing roles, as well as that changes in the steady-state levels or activities of these proteins often affect the alternative splicing of multiple gene transcripts^6^.

Apoptosis—programmed cell death—is a crucial biological process that can be triggered by several stimuli. According to previous studies, a large number of apoptotic genes such as *Homeodomain Interacting Protein Kinase 3* (*HIPK3*), *Second Mitochondria*-*Derived Activator Of Caspases*/*Direct IAP*-*Binding Protein With Low Pi* (*SMAC*/*DIABLO*), and *SURVIVIN* are regulated through alternative splicing^7, 8^. HIPK3, a member of the HIPK family, has been reported to phosphorylate the Fas-associated protein with death domain. Two splicing isoforms of *HIPK3* have been discovered, namely *HIPK3* exon 11-excluded U (−) and *HIPK3* exon 11-included U (+). These HIPK3 isoforms can be regulated by c-Jun NH2-terminal kinase in cancer cells, thus contributing to an increased resistance to Fas receptor-mediated apoptosis^9^. Proteins inhibitory to apoptosis are often characterized by the presence of one or more baculoviral inhibitor-of-apoptosis (IAP) repeat (BIR) domains. SURVIVIN is the smallest member of the IAP family; it functions as a negative regulator of apoptosis through the suppression of CASPASE activation^7^. The antiapoptotic role of SURVIVIN can be regulated by its association with X-linked IAPs (XIAPs) through their conserved BIR domain. The formation of a SURVIVIN/XIAP complex promotes enhanced XIAP stability against ubiquitination/proteasomal destruction and gives rise to the synergistic inhibition of CASPASE-9 activation^10, 11^. In addition, the *SURVIVIN* gene locus encodes multiple genetic splice isoforms with different antiapoptotic properties. For example, SURVIVIN Delta Ex3 is translated from an exon 3 exclusion isoform and is known to act as an adaptor, allowing the formation of a complex between BCL-2 and activated CASPASE-3, and leading to the inhibition of CASPASE-3 activation^12^. By contrast, inhibition of apoptosis by BIR-containing proteins can be antagonized by SMAC/DIABLO. Upon the reception of apoptotic triggers, SMAC/DIABLO is released from mitochondria into the cytosol where it interacts with IAPs and keeps them from inhibiting caspases. Thus, SMAC/DIABLO can abrogate the inhibition of CASPASE-3 and -9 by binding to the BIR2 and BIR3 domains of XIAPs^13^. Alternative splicing also generates isoforms of SMAC/DIABLO with different proapoptotic properties. For example, *SMAC*-*3*, an exon 4 exclusion isoform derived from the *SMAC*-*3* transcript, can accelerate XIAP auto-ubiquitination and destruction. In response to stimuli, SMAC-3 interacts with the BIR2 and BIR3 domains of XIAPs and disrupts the association between XIAP and processed CASPASE-9, causing CASPASE-3 activation and thereby potentiating apoptosis^14^. In humans, aberrant regulation of the alternative splicing of apoptotic gene transcripts is thought to contribute to the development of cancer^15, 16^. These observations imply that modulations of the alternative splicing of apoptotic gene transcripts may offer an effective approach for the treatment of cancer.

Plants that belong to the family *Solanaceae* are rich in withanolides. These compounds have numerous biological activities, including anticancer, anti-inflammatory, antibacterial, and antioxidant activities^17^. In previous studies, we have isolated various withanolides from *Physalis peruviana* and discovered that 4β-Hydroxywithanolide E (4bHWE) has potent cytotoxic activity against cancer cell lines^18^. In the present study, we investigated the novel biological action of 4bHWE, namely the modulation of alternative splicing, in human hepatocellular carcinoma Huh-7 cells and other human cancer cell lines to determine possible therapeutic applications.

## Results

### Effect of 4bHWE on Cell Viability

**Figure 1:**
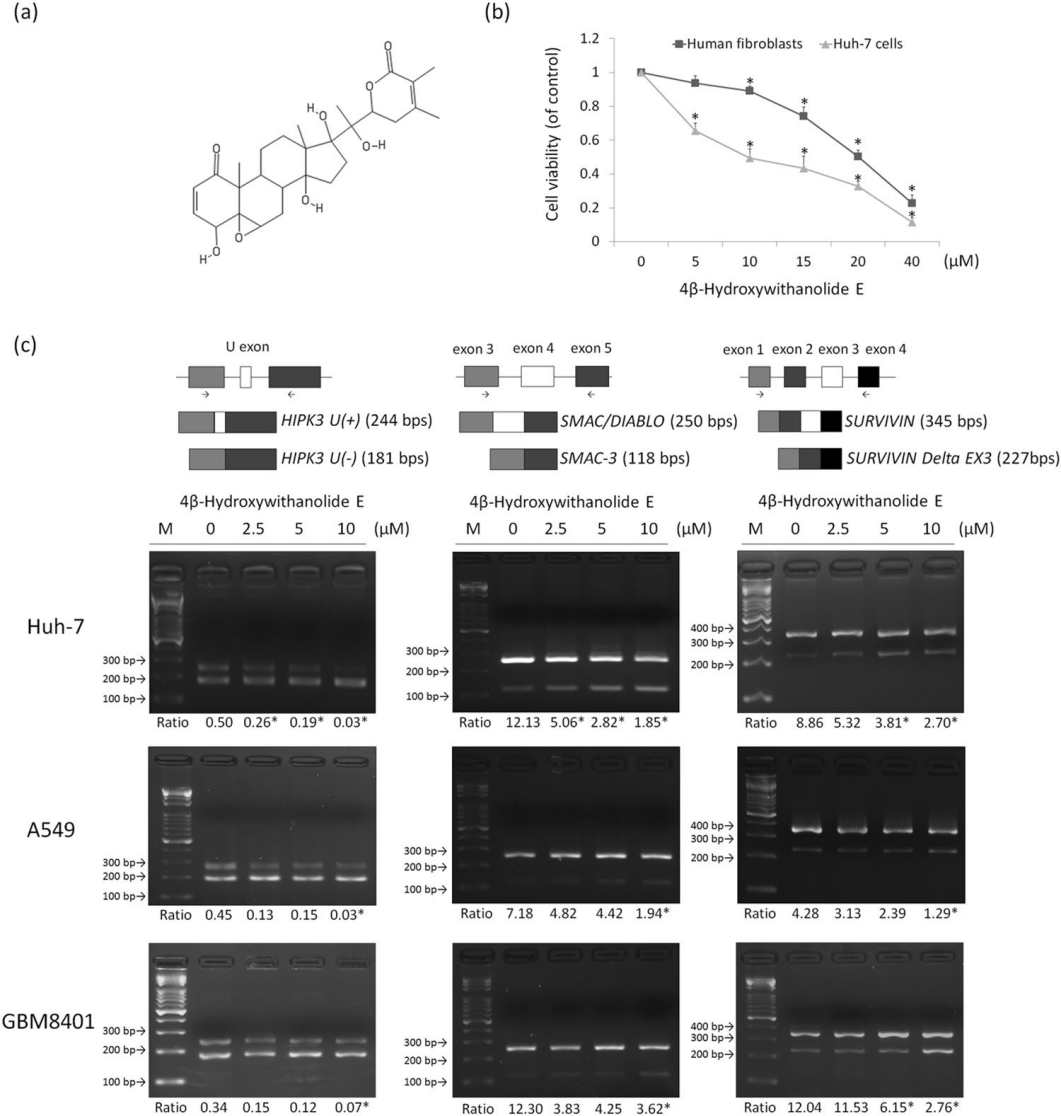
4bHWE modulates the alternative splicing of apoptotic gene transcripts. Cells were treated with 4bHWE at the indicated concentration for 24 h. (**a**) Chemical structure of 4bHWE. (**b**) Cell viability of Huh-7 cells and human fibroblasts were analyzed through MTT assays. (**c**) mRNA was extracted and detected using RT-PCR for the alternative splicing of the *HIPK3*, *SMAC*/*DIABLO*, and *SURVIVIN* transcripts in Huh-7 (second row), A549 (third row), and GBM8401 (fourth row) cells. The splicing isoforms are illustrated in the top row, and their expected PCR products derived through the primers are indicated by arrowheads. The ratios of the densities of the two bands (alternative exon-containing isoforms to alternative exon-lacking isoforms) are presented below each group. Data are the mean of three independent experiments. *p < 0.05 compared with the control. M = marker.

The growth-inhibitory activity of 4bHWE was assessed using an MTT assay. Human hepatocellular carcinoma Huh-7 cells were treated with different concentrations of 4bHWE for 24 h. The results revealed that the 4bHWE-treated Huh-7 cells exhibited a substantial dose-dependent loss of viability (Fig. 1b). The inhibitory effect of 4bHWE on cell survival demonstrated in Huh-7 cells implied that 4bHWE might affect different cell types. Hence, we also treated human fibroblasts with different concentrations of 4bHWE for 24 h. The results revealed that incubating 4bHWE at a concentration of 5 μM did not significantly influence the viability of human fibroblasts, but markedly reduced the viability of Huh-7 cells (Fig. 1b). Moreover, the 50% inhibitory concentrations (IC_50_) of 4bHWE in Huh-7 cells and human fibroblasts were 8.32 μM and 26.42 μM, respectively. Human fibroblasts are more resistant to 4bHWE-induced cell death than are Huh-7 cells. In general, different cell types have different functions and gene expressions, causing the activation of different signal transduction pathways. This may be the cause of the variation in 4bHWE tolerance exhibited by human fibroblasts and Huh-7 cells.

### 4bHWE Modulates the Alternative Splicing of Apoptotic Gene Transcripts

Several reports have concluded that the unbalanced RNA splicing of apoptotic genes is associated with the development of human cancer cells^19^. In this study, we chose *HIPK3*, *SMAC*/*DIABLO*, and *SURVIVIN* as representative genes for screening small molecules and drugs. We discovered that 4bHWE affects the alternative splicing of these three apoptotic gene transcripts in Huh-7 cells. Huh-7 cells treated with 4bHWE exhibited (a) relative maintenance of *HIPK3 U* (−) and a decrease in *HIPK3 U* (+) isoforms, (b) relative maintenance of *SMAC*/*DIABLO* and an increase in *SMAC*-*3* isoforms, and (c) relative maintenance of *SURVIVIN* and an increase in *SURVIVIN Delta EX3* isoforms. These effects of *HIPK3*, *SMAC*/*DIABLO*, and *SURVIVIN* were dose-dependent and detectable after 24 h of incubation with various concentrations of 4bHWE. We further explored the effects of 4bHWE on cell lines derived from different human solid malignant cancers, revealing that 4bHWE had a similar effect on the modulation of the alternative splicing of these three apoptotic gene transcripts in A549 and GBM8401 cancer cells (Fig. 1c).

### 4bHWE Affects the Steady-State Levels of SR Proteins and hnRNPs

**Figure 2:**
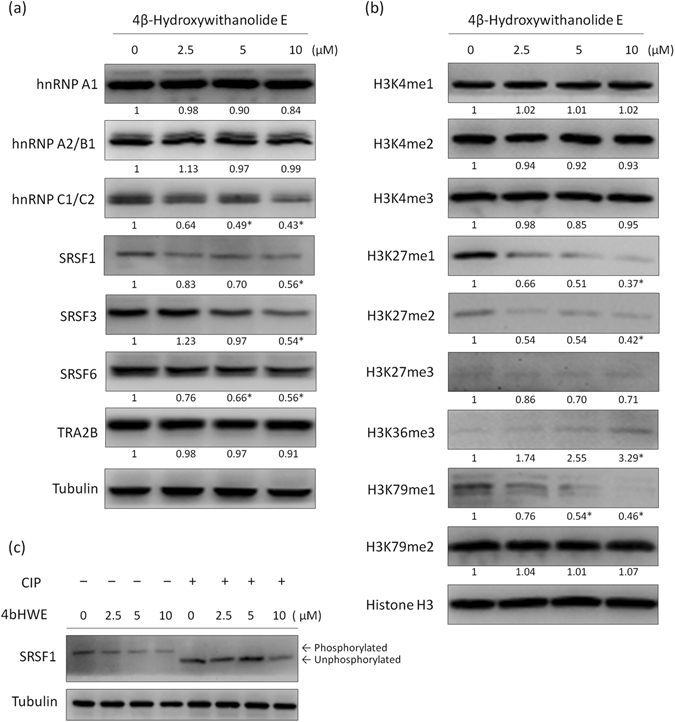
4bHWE affects the splicing factors and histone tail PTMs. Huh-7 cells were treated with the indicated concentration of 4bHWE for 24 h and then harvested. Equal amounts of whole cell lysates (20 μg) were separated using SDS-PAGE and immunoblotted with various antibodies as indicated. (**a**) SR proteins and hnRNPs. (**b**) Histone tail PTMs. Tubulin and Histone H3 are shown as internal standards. The fold-change values are presented below each band. Data are the mean of three independent experiments. *p < 0.05 compared with the control. (**c**) Analysis of the phosphorylation status of SRSF1. Equal amounts of whole cell lysates (20 μg) were incubated with or without three units of CIP at 37 °C for 30 mins, separated by SDS-PAGE, and immunoblotted with various antibodies as indicated. Tubulin is shown as internal standards. Full-length blots are presented in Supplementary Fig. S1.

Because 4bHWE is able to modulate pre-mRNA alternative splicing, we used Western blotting to analyze the steady-state levels of splicing factors. The data demonstrated that hnRNP C1/C2, SRSF1, SRSF3, and SRSF6 were downregulated and that hnRNP A1, hnRNP A2/B1, and TRA2B did not change significantly after 4bHWE treatment (Fig. 2a). These results suggested that 4bHWE may modulate the alternative splicing of *HIPK3*, *SMAC*/*DIABLO*, and *SURVIVIN* transcripts through changes in splicing factors. However, the SRSF1 antibody used in this study was able to recognize both nonphosphorylated and phosphorylated SRSF1. To investigate whether the observed band was a phosphorylated form of SRSF1, Huh-7 cells were treated with 4bHWE for 24 h, and whole cell lysates were incubated with or without calf intestinal alkaline phosphatase (CIP) before sodium dodecyl sulfate polyacrylamide gel electrophoresis (SDS-PAGE) analysis. When CIP was added, SRSF1 was mainly detected as a lower band, indicating that the upper band was the SRSF1 phospho-isoform and the lower band was nonphosphorylated SRSF1 (Fig. 2c). Hence, the results demonstrated that the levels of the SRSF1 phospho-isoform were reduced in 4bHWE treatment. Moreover, because only one band was detected for SRSF1 in 4bHWE-treated cells, the present data suggested that 4bHWE may decrease the steady-state levels of SRSF1, resulting in a decrease in the levels of the SRSF1 phospho-isoform.

### 4bHWE Affects Histone Tail Posttranslational Modifications

Recent studies have revealed crosstalk between histone tail posttranslational modifications (PTMs) and the splicing mechanism, indicating that the patterns of histone tail PTMs affect the splicing outcome by influencing the recruitment of splicing regulators^20^. Therefore, we analyzed the effects of 4bHWE on histone tail PTMs in Huh-7 cells. The data revealed that 4bHWE elevated the levels of H3K36me3 and reduced the levels of H3K27me1, H3K27me2, and H3K79me1, but did not significantly affect the levels of H3K4me1, H3K4me2, H3K4me3, H3K27me3, and H3K79me2 (Fig. 2b). Thus, changes in histone tail PTMs may be involved in 4bHWE-induced splicing regulation.

### Effect of SRSF1 Knockdown or the Reduction of H3K36me3 Levels on the Alternative Splicing of Apoptotic Gene Transcripts

**Figure 3:**
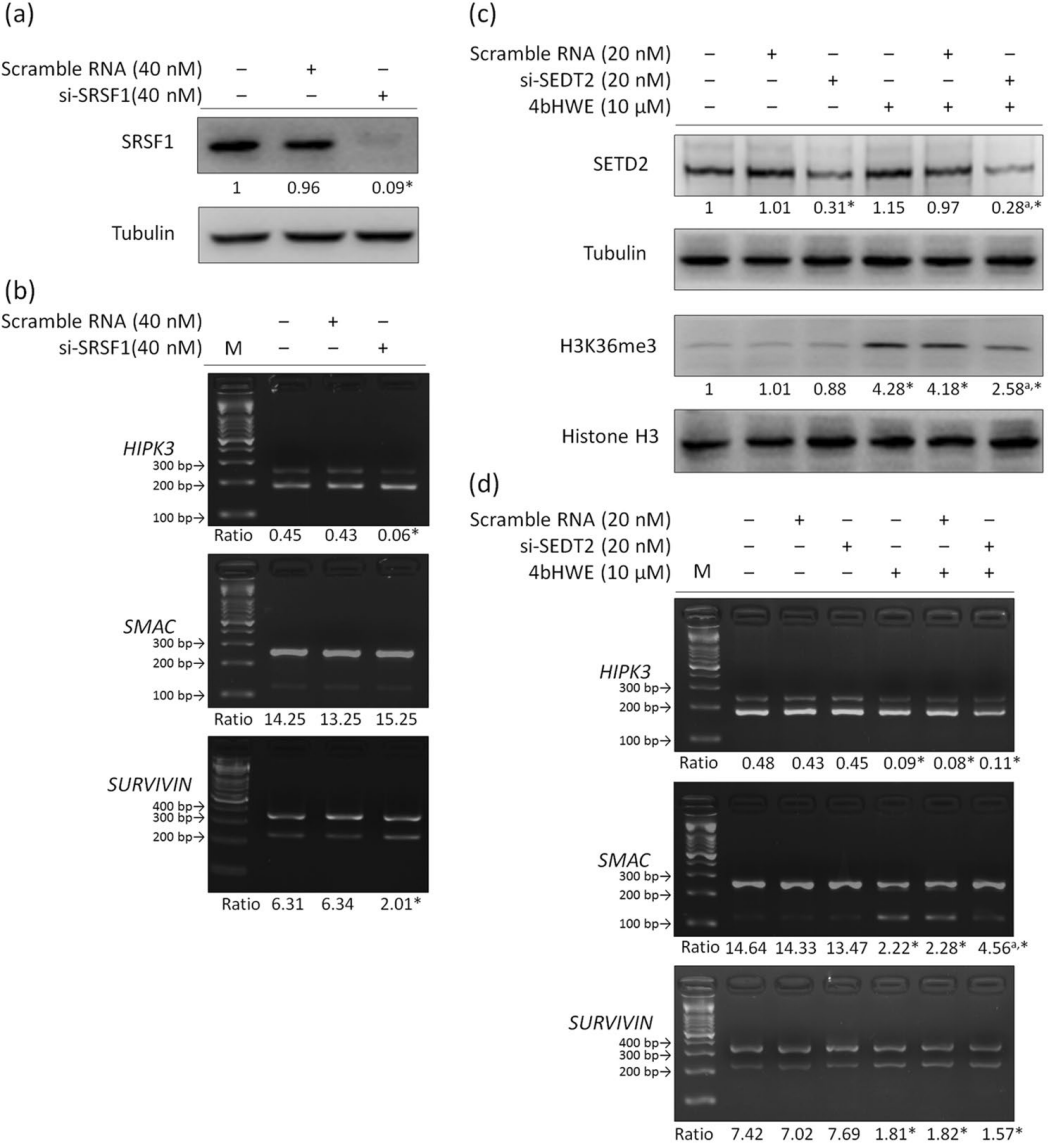
Effect of SRSF1 knockdown or the reduction of H3K36me3 levels on the alternative splicing of apoptotic gene transcripts. (**a**) Huh-7 cells were transfected with 40 nM si-SRSF1 in the SRSF1 knockdown, or 40 nM scramble RNA was used as the negative control for 72 h. The efficiency of the SRSF1 knockdown was assessed through Western blotting. (**b**) mRNAs were extracted and detected using RT-PCR for the alternative splicing of the *HIPK3*, *SMAC*/*DIABLO*, and *SURVIVIN* transcripts. (**c**) Huh-7 cells were transfected with 20 nM si-SETD2 for the reduction of H3K36me3 levels or 20 nM scramble RNA was used as the negative control for 72 h, followed by exposure to 10 μM 4bHWE for 24 h. The efficiency of the SETD2 knockdown and the reduction of H3K36me3 levels was assessed through Western blotting. (**d**) mRNAs were extracted and detected using RT-PCR for the alternative splicing of the *HIPK3*, *SMAC*/*DIABLO*, and *SURVIVIN* transcripts. Western blotting: equal amounts of whole cell lysates (20 μg) were separated using SDS-PAGE and immunoblotted with various antibodies as indicated. Tubulin and Histone H3 are shown as internal standards. The fold-change values are presented below each band. RT-PCR: the ratios of the densities of the two bands (alternative exon-containing isoforms to alternative exon-lacking isoforms) are presented below each group. Data are the mean of three independent experiments. *p < 0.05 compared with the control. ^a^ *p* < 0.05 for the comparison between scramble RNA + 4bHWE and si-SETD2 + 4bHWE. M = marker. Full-length blots are presented in Supplementary Fig. S2.

We determined that 4bHWE may modulate alternative splicing through the regulation of splicing factors and histone tail PTMs. Knockdown experiments were performed to investigate the probable mechanism of modulation by 4bHWE. Huh-7 cells were transfected with 40 nM si-SRSF1. After 72 h, the SRSF1 phospho-isoform levels had decreased by 91% (Fig. 3a). We discovered that SRSF1 knockdown decreased the proportion of *HIPK3 U* (+) and increased the proportion of *SURVIVIN Delta EX3*, but had no significant effect on the alternative splicing of *SMAC*/*DIABLO* transcripts in Huh-7 cells (Fig. 3b). On the other hand, tri-methylated H3K36 is mainly established through SETD2 histone methyltransferase activity^21, 22^. We therefore reduced the levels of H3K36me3 by applying 20 nM si-SETD2 to Huh-7 cells for 72 h and then exposing them to 10 μM 4bHWE for 24 h. The efficiency of the SETD2 knockdown and the reduction of H3K36me3 levels was evaluated using Western blotting (Fig. 3c). The reduction of H3K36me3 levels was demonstrated to partially relieve the effect of 4bHWE on the alternative splicing of *SMAC*/*DIABLO* transcripts, but not its effect on the alternative splicing of *HIPK3* and *SURVIVIN* transcripts (Fig. 3d). Thus, these data suggested that changes in the levels of SRSF1 phospho-isoform and H3K36me3 may be involved in 4bHWE-induced splicing regulation.

### Effect of 4bHWE on AKT Kinase, SRPK1, SRPK2, and the Phosphorylation of Protein Phosphatase-1

**Figure 4:**
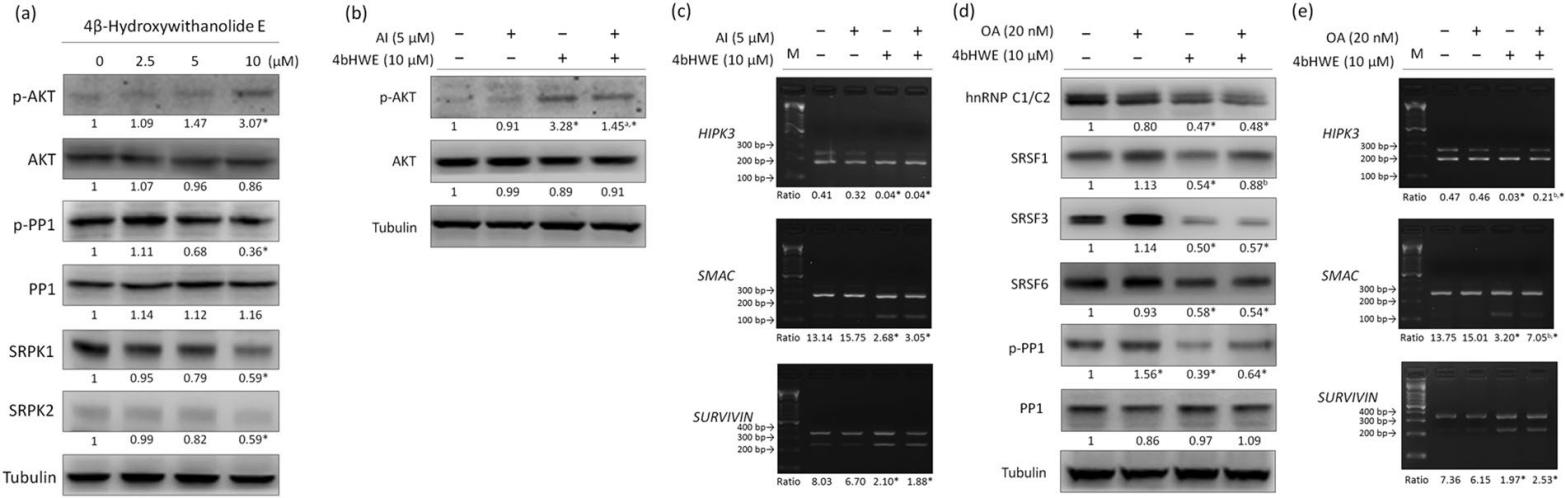
Effect of 4bHWE on AKT kinase, SRPK1, SRPK2, and PP1. (**a**) Huh-7 cells were treated with the indicated concentration of 4bHWE for 24 h. The proteins were analyzed through Western blotting. (**b**) and (**c**) Huh-7 cells were treated with 5 μM AKT inhibitor for 1 h and then exposed to 10 μM 4bHWE for 24 h. The proteins and mRNA were analyzed through Western blotting and RT-PCR, respectively. (**d**) and (**e**) Huh-7 cells were treated with 20 nM okadaic acid for 1 h and then exposed to 10 μM 4bHWE for 24 h. The proteins and mRNA were analyzed through Western blotting and RT-PCR, respectively. Western blotting: equal amounts of whole cell lysates (20 μg) were separated using SDS-PAGE and immunoblotted with various antibodies as indicated. Tubulin and Histone H3 are shown as internal standards. The fold-change values are presented below each band. RT-PCR: mRNA was extracted and detected for the alternative splicing of the *HIPK3*, *SMAC*/*DIABLO*, and *SURVIVIN* transcripts. The ratios of the densities of the two bands (alternative exon-containing isoforms to alternative exon-lacking isoforms) are presented below each group. Data are the mean of three independent experiments. *p < 0.05 compared with the control. ^a^ *p* < 0.05 for the comparison between 4bHWE and AKT inhibitor + 4bHWE. ^b^ *p* < 0.05 for the comparison between 4bHWE and okadaic acid + 4bHWE. AI = AKT inhibitor; OA = okadaic acid; M = marker. Full-length blots are presented in Supplementary Fig. S3.

Previous studies have highlighted the ability of AKT kinase, protein phosphatase-1 (PP1), and SR protein-specific kinases (SRPKs) to directly or indirectly regulate the function of numerous splicing factors and modulate the alternative splicing of several RNA transcripts^23, 24^. Therefore, we investigated the effect of 4bHWE on AKT kinase, SRPK1, SRPK2, and PP1 in Huh-7 cells. The results demonstrated that 4bHWE reduced the steady-state levels of SRPK1 and SRPK2, activated AKT kinase by increasing the phosphorylation level of AKT (Ser473), and activated PP1 by reducing the phosphorylation level of PP1 (Thr320; Fig. 4a). To investigate whether 4bHWE-activated AKT kinase regulates alternative splicing, Huh-7 cells were cultured in the presence of a 5 μM AKT inhibitor to inhibit AKT kinase activity before 4bHWE treatment, and the efficiency of the reduction of the phosphorylation level of AKT (Ser473) was validated by Western blotting (Fig. 4b). However, the AKT inhibitor was revealed to have no significant effect on the alternative splicing of the *HIPK3*, *SMAC*/*DIABLO*, and *SURVIVIN* transcripts in 4bHWE-treated cells (Fig. 4c). Additionally, we pretreated Huh-7 cells with 20 nM okadaic acid to inhibit PP1 activity^25^ prior to 4bHWE treatment. The okadaic acid pretreatment partially relieved the effects of 4bHWE on the alternative splicing of the *HIPK3* and *SMAC*/*DIABLO* transcripts (Fig. 4e) and the levels of SRSF1 phospho-isoform (Fig. 4d). However, okadaic acid had no definitive effect on the alternative splicing of the *SURVIVIN* transcripts (Fig. 4e) or the steady-state levels of hnRNP C1/C2, SRSF3, and SRSF6 in 4bHWE-treated cells (Fig. 4d). These results implied that PP1 may partly mediate the effects of 4bHWE on the alternative splicing of apoptotic gene transcripts.

### 4bHWE Affects the Genome-Wide Alternative Splicing Detected by RNA Sequencing

**Figure 5:**
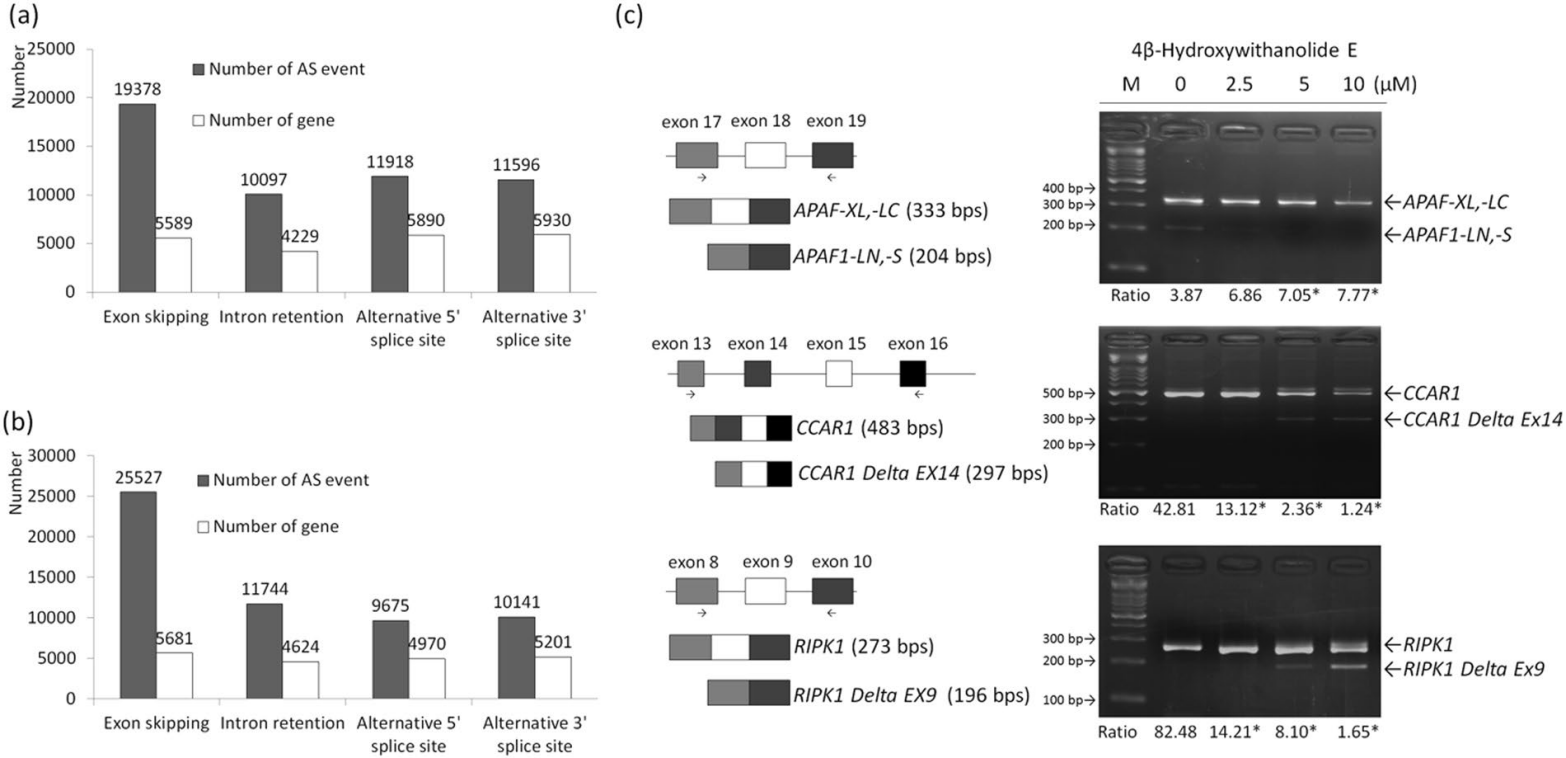
4bHWE affects genome-wide alternative splicing detected using RNA sequencing. Huh-7 cells were treated with (**a**) 0.1% DMSO and (**b**) 10 μM 4bHWE for 24 h prior to RNA sequencing. Grey bars indicate the number of alternative splicing events and white bars indicate the number of genes in which alternative splicing events occurred. The number shown above the bar is the event. This experiment was performed once. (**c**) Huh-7 cells were treated with the indicated concentration of 4bHWE for 24 h and then harvested. mRNAs were extracted and detected using RT-PCR for the alternative splicing of *APAF1*, *CCAR1*, and *RIPK1* transcripts. The splicing isoforms are illustrated in the left column, and their expected PCR products using the primers are indicated by arrowheads. The ratios of the densities of the two bands (alternative exon-containing isoforms to alternative exon-lacking isoforms) are presented below each group. Data are the mean of three independent experiments. *p < 0.05 compared with the control. M = marker.

On the basis of these results, we determined that 4bHWE can modulate the steady-state levels of SR proteins and hnRNPs. This suggested that 4bHWE has substantial effects on pre-mRNA alternative splicing. Therefore, we analyzed the global genes with altered alternative splicing in 4bHWE-treated Huh-7 cells through RNA sequencing, and we discovered that 4bHWE can affect the alternative splicing of multiple gene transcripts and the frequency of alternative splicing (Fig. 5a and b). A total of 8928 gene transcripts with altered alternative splicing were detected after the 4bHWE treatment. Gene function analysis indicated that these genes belong to numerous categories, including apoptosis, cell adhesion, cell cycle, cytoskeleton remodeling, DNA damage, gene expression, and survival. We randomly selected three apoptosis-related genes, namely apoptotic peptidase activating factor 1 (*APAF1*), cell cycle and apoptosis regulator 1 (*CCAR1*), and receptor-interacting protein kinase 1 (*RIPK1*), and further confirmed the changes in alternative splicing through the reverse transcription polymerase chain reaction (RT-PCR). Distinct alternative splicing patterns were detected after treatment with 4bHWE (Fig. 5c). Huh-7 cells treated with 4bHWE exhibited (a) decreases in both the *APAF1*-*XL*,-*LC* and *APAF1*-*LN*,-*S* isoforms, (b) a relative decrease in *CCAR1* and increase in *CCAR1 Delta Ex14* isoforms, and (c) the relative maintenance of *RIPK1* and increase of *RIPK1 Delta Ex9* isoforms. Therefore, 4bHWE had a genome-wide influence on pre-mRNA alternative splicing. Moreover, Sveen *et al*. demonstrated that aberrant splicing events have the potential to function as cancer biomarkers and therapeutic targets^16^. In our study, we also discovered that 4bHWE can modulate the alternative splicing of numerous candidate biomarker transcripts such as *AIMP2*, *BCL2L11*, *BIRC5*, *CASP3*, *CEACAM1*, *CPE*, *FGFR2*, *FN1*, *FPGS*, *HIF1A*, *KLF6*, *MCL1*, *MDM2*, *MKNK2*, *TERT*, and *VEGFA*. This suggests that 4bHWE may have potential for use in cancer treatment.

### 4bHWE Arrested the Cell Cycle at the G_2_/M Phase and Induced Apoptosis

**Figure 6:**
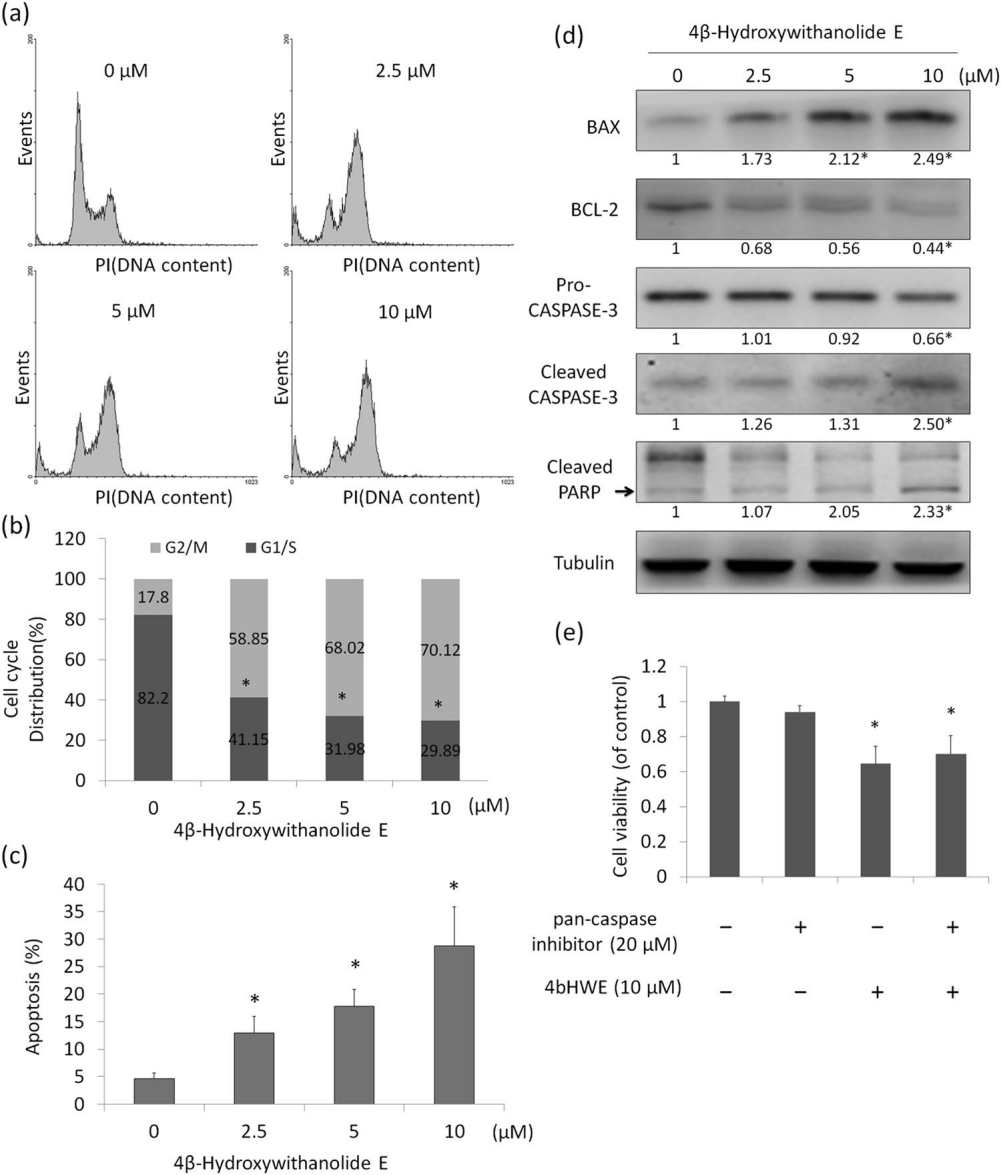
4bHWE arrested the cell cycle at the G_2_/M phase and induced apoptosis. Huh-7 cells were treated with 4bHWE at the indicated concentration for 24 h and then stained with PI. (**a**) DNA content was analyzed using flow cytometry. (**b**) Histogram of the cell cycle distribution. (**c**) Histogram of the apoptotic sub-G_1_ population. (**d**) Huh-7 cells were treated with the indicated concentration of 4bHWE for 24 h and then harvested. Equal amounts of whole cell lysates (20 μg) were separated using SDS-PAGE and immunoblotted with various antibodies as indicated. Tubulin is shown as an internal standard. The fold-change values are presented below each band. (**e**) Effect of a pan-caspase inhibitor on 4bHWE-induced apoptosis in Huh-7 cells. Cells were pretreated with 20 μM pan-caspase inhibitor for 1 h, and then treated with 10 μM 4bHWE for 24 h. Cell viability was determined using MTT assay. Data are the mean of three independent experiments. *p < 0.05 compared with the control. Full-length blots are presented in Supplementary Fig. S4.

Our data indicated that 4bHWE affects the alternative splicing of apoptosis-related gene transcripts, suggesting that 4bHWE exerts a potent effect on cellular apoptosis mechanisms. Therefore, we investigated the effects of 4bHWE on cell cycle and viability through a flow cytometric analysis. The results revealed that 4bHWE significantly increased the population of the G_2_/M phase of the cell cycle (Fig. 6a and b). The treatment of Huh-7 cells with 0, 2.5, 5, and 10 μM 4bHWE resulted in an accumulation of G_2_/M phase cells corresponding to 17.8%, 58.8%, 68.0%, and 70.1%, respectively (Fig. 6b). In addition, an increasing number of apoptotic sub-G_1_ phase cells were detected among 4bHWE-treated cells. Treatment of cells with 2.5, 5, and 10 μM 4bHWE increased the percentage of apoptotic sub-G_1_ phase cells from 4.5% to 12.9%, 17.7%, and 28.7%, respectively (Fig. 6c). We further examined the steady-state levels of the apoptosis-related proteins, finding that 4bHWE increased the steady-state levels of BAX, the active form of CASPASE-3, and the cleavage of poly ADP-ribose polymerase; 4bHWE also reduced the steady-state levels of BCL-2 (Fig. 6d). Therefore, 4bHWE affected cell cycle progression and apoptosis. Moreover, we pretreated Huh-7 cells with 20 μM pan-caspase inhibitor to inhibit caspase activity prior to 4bHWE treatment. However, the results revealed that 4bHWE-induced apoptosis was not significantly reversed by the pan-caspase inhibitor (Fig. 6e). This implied that 4bHWE-induced cell death not only involved the apoptotic signaling pathway but also other death-related pathways.

### Inhibitory Effect of 4bHWE on Tumor Growth in a Xenograft Animal Model

**Figure 7:**
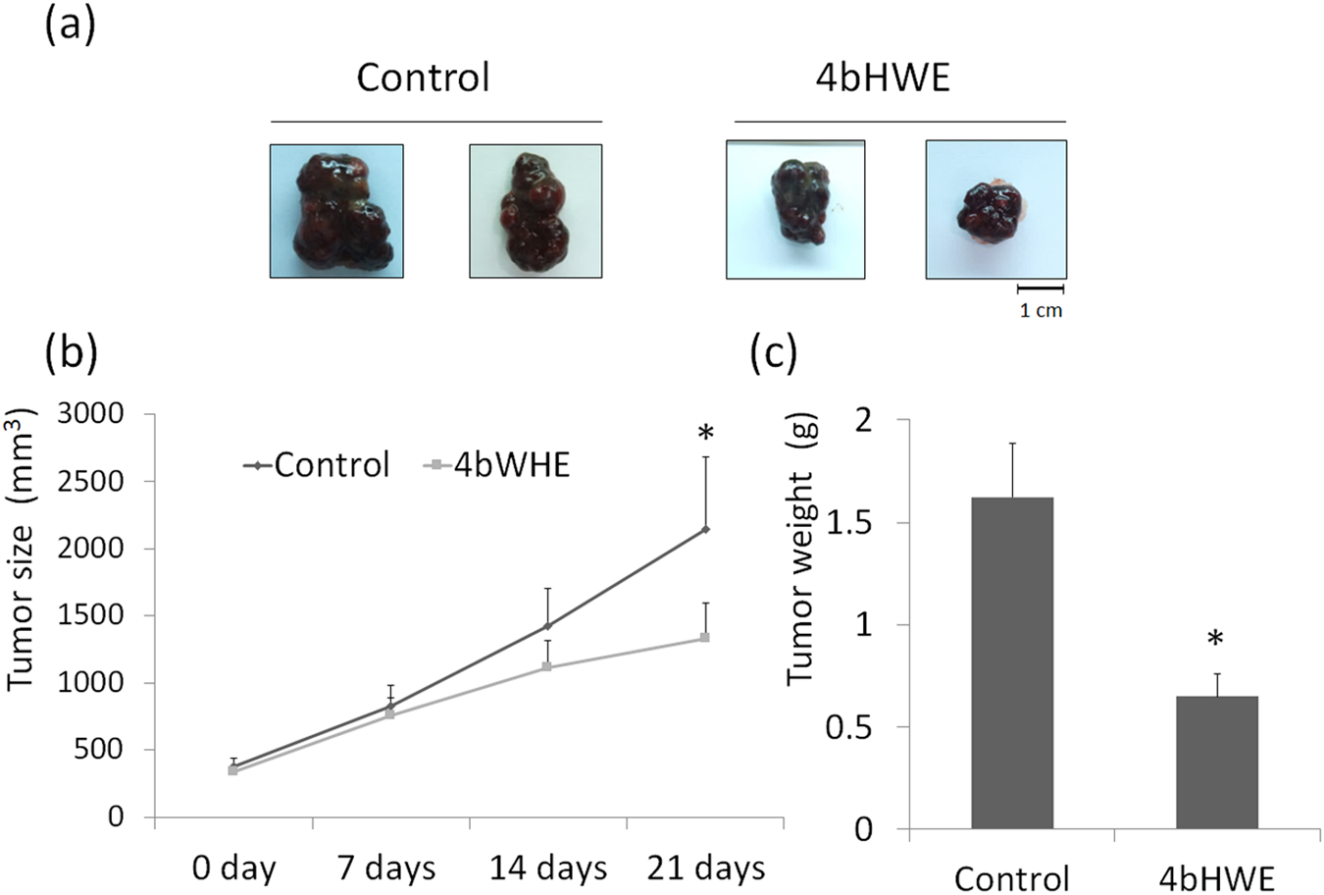
Inhibitory effect of 4bHWE on tumor growth in a xenograft animal model. Male nude mice bearing Huh-7 cell tumors were treated with a solvent (control) or 4bHWE (11.6 μg/10 g) for 21 days. (**a**) Representative picture of tumor growth in xenograft nude mice administered solvent (left) and 4bHWE (right). Scale bar = 1 cm. (**b**) Tumor volumes were measured after therapy was initiated. (**c**) Histogram of tumor weight. *p < 0.05 compared with the control.

Because 4bHWE inhibited the growth of cancer cells, evaluating the therapeutic effects of 4bHWE *in vivo* was useful. Hence, Huh-7 cells were inoculated subcutaneously in the right flanks of several nude mice. When the tumors reached a size of approximately 300 mm^3^, the nude mice were administered 4bHWE or a control solvent every other day. After 21 days of treatment, the tumors in the 4bHWE-treated group were clearly smaller than those in the control group (Fig. 7a). The average tumor size in the control group was 2275.5 mm^3^, whereas the average tumor size in the 4bHWE-treated group was 1332.8 mm^3^ (Fig. 7b). At the end of treatment, the tumor tissue was isolated and weighed. The tissue extracted from the 4bHWE-treated group weighed considerably less than that extracted from the control group (0.64 vs 1.62 g; Fig. 7c).

## Discussion

In this study, we initially observed that 4bHWE affected the modulation of the alternative splicing of the *HIPK3*, *SMAC*/*DIABLO*, and *SURVIVIN* transcripts. In subsequent experiments, we discovered that the levels of SRSF1 phospho-isoform had decreased and the levels of H3K36me3 had increased in 4bHWE-treated Huh-7 cells. H3K36me3 has been suggested as an epigenetic mark for exons in various species^26, 27^. The H3K36me3 mark can be bound by PC4 and SFRS1-interacting protein 1, and it recruits SRSF1 to its target RNA, thereby promoting the inclusion of an alternative exon. Another H3K36me3-binding protein, namely MORF-related gene 15, can also affect alternative splicing by recruiting the splicing silencer polypyrimidine tract-binding protein to repress the inclusion of an alternative exon^28^. In this study, knockdown experiments demonstrated that the decrease in H3K36me3 levels partially relieved the effect of 4bHWE on the alternative splicing of *SMAC*/*DIABLO* transcripts. This suggested that the splicing site selection of *SMAC*/*DIABLO* may be mediated by changes in the level of H3K36me3 in 4bHWE-treated cells. By contrast, SRSF1 knockdown reduced the proportion of *HIPK3 U* (+) and increased the proportion of *SURVIVIN Delta EX3* in Huh-7 cells. These splicing patterns of SRSF1 knockdown were similar to those produced by 4bHWE treatment, implying that 4bHWE may regulate the alternative splicing of *HIPK3* and *SURVIVIN* transcripts through SRSF1 modulation. However, we also discovered that 4bHWE reduced the steady-state levels of hnRNP C1/C2, SRSF3, and SRSF6 as well as the levels of H3K27me1, H3K27me2, and H3K79me1 in Huh-7 cells. A detailed examination will still be required for exploring the roles of these splicing factors and histone tail PTMs in 4bHWE-induced alternative splicing.

Several reports have suggested a key role for the phosphorylation status of splicing factors in their regulation of alternative splicing^23^. We extended our study to examine whether AKT kinase and PP1 influence alternative splicing factors during 4bHWE treatment by pretreating cells with AKT inhibitor or okadaic acid. The results demonstrated that PP1 may partly mediate the effects of 4bHWE on the alternative splicing of *HIPK3* and *SMAC*/*DIABLO* transcripts. However, okadaic acid is not only a potent inhibitor of PP1, but also inhibits a range of other phosphatases^29, 30^. The inhibition of PP1 may not have been the only cause of the observed results; thus, further studies are required to investigate the role of PP1 in 4bHWE-induced alternative splicing. On the other hand, studies have reported that AKT kinase acts as a regulator of alternative splicing and SR protein phosphorylation. AKT kinase not only directly phosphorylates multiple splicing factors (e.g., SRSF1, SRSF5, and SRSF7)^31–33^ but also indirectly regulates the function of SR proteins by binding and inducing the autophosphorylation of SRPKs. AKT kinase switches SRPKs from Hsp70- to Hsp90-containing complexes, thereby increasing SRPK nuclear translocation and the phosphorylation level of SR proteins. In this AKT-SRPKs-SR signal transduction pathway, Hsp70 plays an essential role in the inhibition of the nuclear import of SRPKs. By contrast, Hsp90 can remove Hsp70 from SRPK-containing complexes to facilitate the nuclear import of SRPKs^24^. In our previous studies, 4bHWE increased the steady-state levels of Hsp70 and inhibited Hsp90 function through the degradation of Hsp90 client proteins^34^. In the current study, we observed the activation of AKT kinase and reduced the steady-state levels of SRPK1 and SRPK2 in 4bHWE-treated cells. The inhibition of the AKT kinase signaling cascade by AKT inhibitors did not significantly mitigate the effects of 4bHWE on the alternative splicing of the *HIPK3*, *SMAC*/*DIABLO*, and *SURVIVIN* transcripts. This suggests that abnormal steady-state levels of Hsp70, Hsp90, and SRPKs may cause the failure of the nuclear translocation of SRPKs and the phosphorylation of SR proteins, resulting in the improper regulation of the alternative splicing of gene transcripts in 4bHWE-treated cells.

Apoptosis is known to occur in various biological processes. Disarrangement of the apoptotic signaling pathway is related to various human diseases, including cancer, and failure to activate this pathway can cause cancer cells to become resistant to the cytotoxic effects of numerous chemotherapy drugs. Accordingly, shifting splicing toward proapoptotic isoforms presents a notable opportunity for cancer therapy. We discovered that 4bHWE decreases the proportion of *HIPK3 U* (+) and increases the proportion of *SMAC*-*3* and *SURVIVIN Delta Ex3* in human cancer cell lines. These findings indicate that these apoptosis-related genes may participate in 4bHWE-induced apoptosis. Moreover, we observed through RNA sequencing that the splicing of three other apoptosis-related gene transcripts, namely *APAF1*, *CCAR1*, and *RIPK1*, was altered during 4bHWE treatment. APAF1 is a critical member of the apoptosome, which regulates the cytochrome c-dependent autocatalytic activation of proCASPASE-9 and the maintenance of processed CASPASE-9 activity^35^. *APAF1* can generate two transcripts: the proapoptotic and prosurvival splice isoforms of *APAF1* (*APAF1*-*LN*,-*S* and *APAF1*-*XL*,-*LC*, respectively). We found that the proportion of *APAF1*-*LN*,-*S* and *APAF1*-*XL*,-*LC* had all decreased in the 4bHWE-treated cells, indicating that changes in the proportion of APAF1 isoforms may be involved in 4bHWE-induced apoptosis. CCAR1 was originally identified as a perinuclear phosphoprotein that serves as a coactivator of nuclear receptors, anaphase promoting complex/cyclosome E3 ligase, and p53 to regulate cell growth and apoptosis. Recent studies have indicated that the inhibition of CCAR1 through CARP-1 functional mimetics (CFMs) can stimulate apoptosis and reduce invasion, migration, and colony formation in human medulloblastoma cells^36, 37^. In the current study, a decreased proportion of *CCAR1* and an increased proportion of *CCAR1 Delta Ex14* were identified in the 4bHWE-treated cells. Thus, 4bHWE may partially regulate the apoptosis signaling pathway through the modulation of the alternative splicing of *CCAR1* transcripts. RIPK1 is a protein Ser/Thr kinase with numerous physiological functions, including innate immunity and both the positive and negative control of apoptosis and programmed necrosis (necroptosis). During the binding of tumor necrosis factor (TNF) to TNF receptor 1 (TNFR1), complex I—which consists of RIPK1, TNFR1-associated death domain protein, TNFR-associated factor 2, and cellular inhibitor-of-apoptosis protein 1 (cIAP1)—can form at the cytoplasmic membrane, allowing cIAP1 to add K63-linked polyubiquitin chains to RIPK1. The ubiquitination of RIPK1 functions as a scaffold for the recruitment of transforming growth factor beta activated kinase-1 and IκB kinase complex in the nuclear factor κB and mitogen-activated protein kinase survival pathways^38, 39^. *RIPK1* can be translated into 671 amino acids; however, the *RIPK1 Delta Ex9* loses exon 9, causing a frameshift and a premature stop at codon 298. The structural change of RIPK1 Delta Ex9 may affect the functions of complex I and further inhibit the survival pathways in 4bHWE-treated cells. These findings clearly indicate that 4bHWE may play a role in the generation of a proapoptotic or antisurvival phenotype by modulating the alternative splicing of various apoptosis-related genes, yielding improved sensitization of cancer cells to chemotherapy or radiation and representing a new target for anticancer therapies.

**Figure 8:**
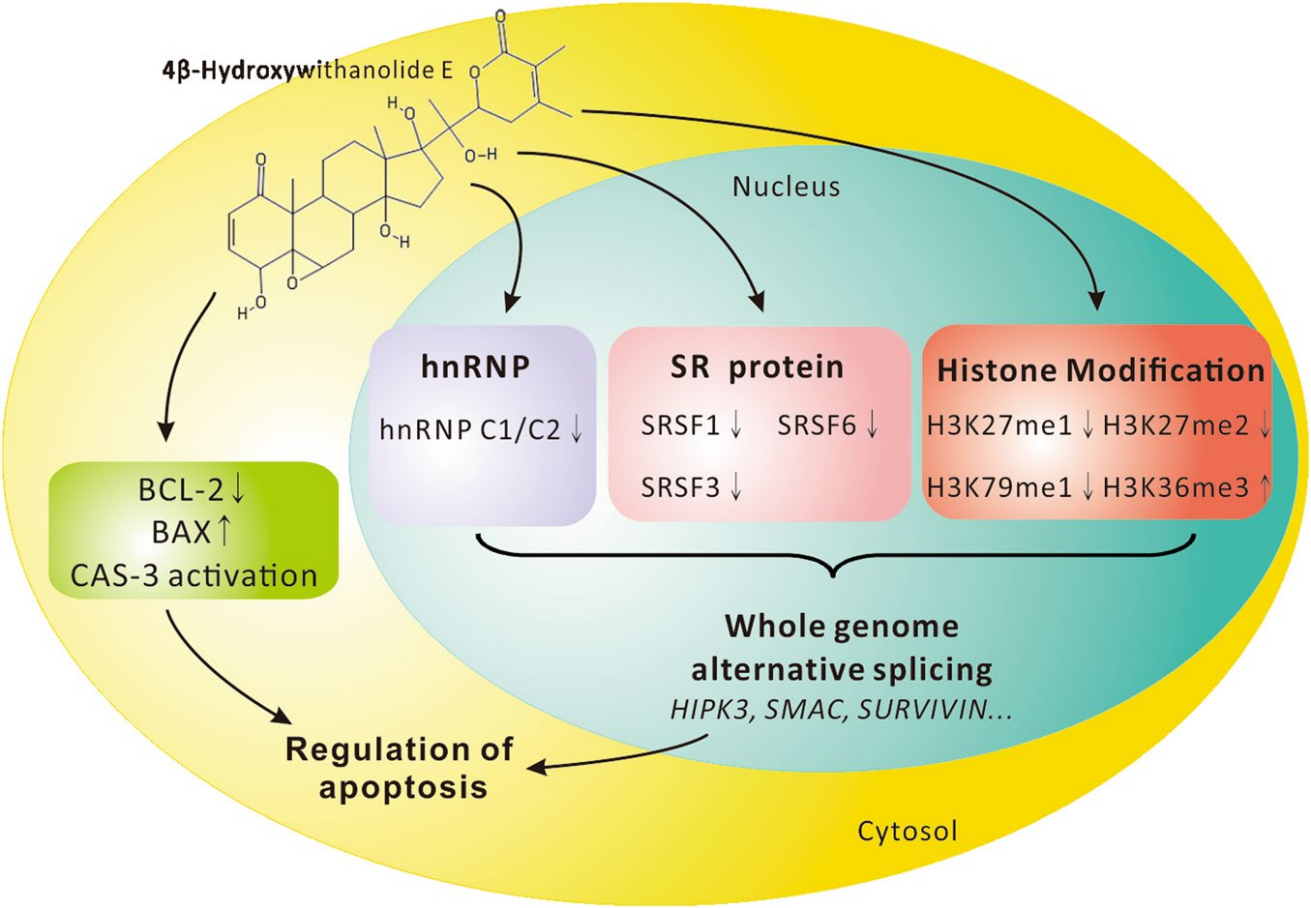
Hypothetical schematic diagram of 4bHWE-induced alternative splicing in Huh-7 cells.

Withanolides are a group of natural steroidal lactones with potent cytotoxic activity against human tumors. Studies on the anticancer properties of withanolides have been performed for several years^40^; however, their anticancer activity remains unclear. This study was the first to demonstrate that 4bHWE affects alternative splicing through the modulation of splicing factors and histone tail PTMs, thus causing apoptosis (Fig. 8). Our findings offer a novel view of the antitumor mechanisms of 4bHWE.

## Materials and Methods

### Reagents

As shown in Fig. 1a, 4bHWE, which was provided by Dr. Yang-Chang Wu (School of Chinese Medicine, College of Chinese Medicine, China Medical University, Taichung, Taiwan), was dissolved in dimethyl sulfoxide (DMSO) to produce 10 mM stock solutions. AKT inhibitor (Cat. No. 124005) and pan-caspase inhibitor Z-VAD-FMK (Cat. No. 219007) were obtained from Millipore. Okadaic acid (Cat. No. O9381) was obtained from Sigma. CIP was obtained from New England Biolabs.

### Antibodies

Antibodies were purchased from the following companies: anti-H3K27me1, H3K27me3, H3K79me1, H3K79me2, histone H3, SETD2, and tubulin from Abcam; anti-hnRNP A2/B1 from Acris; anti-SRPK1 and SRPK2 from BD Biosciences; anti-AKT, H3K27me2, PP1, phospho-PP1 at Thr320, phospho-AKT at Ser473, and cleaved-CASPASE-3 from Cell Signaling Technology; anti-H3K4me1, H3K4me2, H3K4me3, and H3K36me3 from Millipore; anti-pro CASPASE-3 from GeneTex; anti-hnRNP C1/C2, BAX, BCL-2, and PARP from Santa Cruz Biotechnology; anti-hnRNP A1, SRSF6, and TRA2B from Sigma; and anti-SRSF1 and SRSF3 from Zymed.

### Cell Culture and Gene Knockdown

A human fibroblast cell line (established from normal human skin, 27 years old, male, Asian) was kindly provided by Dr. Chung-Hsing Chang (School of Medicine, College of Medicine, China Medical University, Taichung, Taiwan), and the A549, GBM8401, and Huh-7 cell lines were obtained from the Bioresource Collection and Research Center. Cells were maintained in Dulbecco’s Modifed Eagle’s medium that was supplemented with 10% fetal bovine serum and antibiotics (100 U/mL penicillin and 100 μg/mL streptomycin) at 37 °C in a humidified atmosphere of 5% CO_2_. To perform SRSF1 or SEDT2 knockdown, antisense oligonucleotide sequences of SRSF1, SEDT2, or a scrambled control (Supplementary Table S1) were transfected into cells using Lipofetamine RNAiMAX (Invitrogen) according to the manufacturer’s protocol.

### Cell Growth Inhibition Assay

Cell viability was determined using the MTT (3-(4,5-dimethylthiazol-2-yl)-2,5-diphenyltetrazolium bromide) assay; the results are presented as a percentage of the control results. In the MTT assay, 10 μL of MTT (5 mg/mL) dye was directly added to the cell cultures. The medium was removed after 2 h, and the cells were then lysed with 100 μL of DMSO. The absorbance at 565 nm was measured using a microplate reader.

### Protein Extracts and Western Blotting

Total cellular proteins were obtained using a cell lysis solution (20 mM Tris-HCl, pH 7.5, 150 mM NaCl, 1 mM EDTA, 1% Nonidet P-40, 1% sodium deoxycholate, 2.5 mM sodium pyrophosphate, 1 mM b-glycerophosphate, 1 mM Na_3_VO_4_, and 1 µg/mL leupeptin). Proteins were extracted from the experimental and control samples and analyzed using SDS-PAGE as follows: After electrophoresis, proteins were transferred from the gel onto polyvinylidene fluoride membranes (Millipore). The membranes were blocked with 5% bovine serum albumin (Santa Cruz Biotechnology) and then exposed overnight at 4 °C to the appropriate concentrations of primary antibodies, followed by horseradish peroxidase-conjugated secondary antibody for detection by an enzyme chemiluminescence kit (Amersham Inc.). The quality of each group was measured using Gel-Pro Analyzer 4.0 and compared with that of the control group by calculating a percentage.

### Reverse Transcription PCR Analysis

We extracted mRNA from the cells using a TurboCapture 8 mRNA kit (Qiagen), and then converted it into cDNA using M-MLV reverse transcriptase (Promega) according to the manufacturer’s instructions. PCR was performed using specific pairs of primers (Supplementary Table S2). The ratio of the densities of the two bands (alternative exon-containing isoforms to alternative exon-lacking isoforms) was determined using Gel-Pro Analyzer 4.0.

### Flow Cytometry

To analyze the cell cycle distribution, cells were collected and fixed in 70% (v/v) ethanol at 4 °C for 30 min. After fixation, the cells were treated with 0.2 mL of the DNA extraction buffer (0.2 M Na_2_HPO_4_ and 0.1 M citric acid buffer; pH 7.8) for 30 min, centrifuged, and then resuspended in 1 mL of propidium iodide (PI) staining buffer (0.1% TritonX-100, 100 μg/mL of RNase A, and 500 μg/mL of PI in phosphate buffered saline) at 37 °C for 30 min. Cells were detected using a flow cytometer and analyzed using BD FACSDiVa software (BD Biosciences).

### RNA Sequencing

Samples were prepared using an mRNA-seq sample kit (Illumina) according to the manufacturer’s instructions. The raw sequencing data has been deposited in the National Center for Biotechnology Information’s Gene Expression Omnibus (GEO) and can be obtained through GEO Series with the access number GSE79884.

### Xenograft Animal Model of Human Hepatocellular Carcinoma Huh-7 Cells

Six-week-old male BALB/cAnN.Cg-Foxn1^nu^/CrlNarl mice were obtained from the National Laboratory Animal and Research Center (Taipei, Taiwan). Aliquots of 5 × 10^6^ Huh-7 cells were subcutaneously injected into the right flank of each mouse, and after 4 weeks (when tumors with diameters of approximately 8–10 mm, equal to approximately 300 mm^3^ volume, had developed), the mice were randomly divided into the control (n = 6) or 4bHWE treatment (n = 6) groups. The mice were subcutaneously injected every other day with 0.1 mL of solvent (normal saline containing 1% DMSO) for the control group or 11.6 μg/10 g of 4bHWE for the 4bHWE treatment group. The tumor size was measured every 7 days with a caliper according to the following formula: volume (mm^3^) = length × width^2^ × 0.5. All animal experiments were performed in accordance with the guidelines established by the Institutional Animal Care and Use Committee (IACUC) of China Medical University (CMU). All animals were housed in the Laboratory Animal Center of CMU under a 12 h light/dark (08:00/20:00) cycle with free access to food and water. The mice were sacrificed using CO_2_ and the tumor tissues were subsequently harvested. All breeding and subsequent use of animals in this study, including sacrifice, was approved by the IACUC of CMU. The IACUC approval number was 103-264-B.

### Statistical Analysis

The differences between the control and the 4bHWE-treated group were analyzed using Student’s *t* tests, with a probability of less than 5% (*p* < 0.05) indicating significance.

## Electronic supplementary material

SUPPLEMENTARY INFO
