# Supplementary material for: 4β-Hydroxywithanolide E Modulates Alternative Splicing of Apoptotic Genes in Human Hepatocellular Carcinoma Huh-7 Cells
Source: Sci Rep. 2017 Aug 4;7:7290. doi: 10.1038/s41598-017-07472-6 (PMC5544667; doi:10.1038/s41598-017-07472-6)
Supplement: Supplementary file 1 — SUPPLEMENTARY INFO [file 41598_2017_7472_MOESM1_ESM.pdf]

4β-Hydroxywithanolide E Modulates Alternative Splicing of Apoptotic Genes in Human Hepatocellular Carcinoma Huh-7 Cells

Chien-Chin Lee[1], Wen-Hsin Chang[2], Ya-Sian Chang[1,3], Ting-Yuan Liu[4], Yu-Chia Chen[4], Yang-Chang Wu[5,6,7,*],Jan-Gowth Chang[1,3,8,*]

[1] Epigenome Research Center, China Medical University Hospital, Taichung, Taiwan.

[2]Department of Primary Care Medicine, Taipei Medical University Hospital, Taipei, Taiwan.

[3]Department of Laboratory Medicine, China Medical University Hospital, Taichung, Taiwan.

[4]Graduate Institute of Medicine, Kaohsiung Medical University Hospital, Kaohsiung, Taiwan.

[5]School of Pharmacy, College of Pharmacy, China Medical University, Taichung, Taiwan.

[6]Chinese Medicine Research and Development Center, China Medical University Hospital, Taichung, Taiwan.

[7]Center for Molecular Medicine, China Medical University Hospital, Taichung, Taiwan.

[8]School of Medicine, China Medical University, Taichung, Taiwan.

* Corresponding authors: Jan-Gowth Chang, Epigenome Research Center, China Medical University Hospital, 2 Yuh-Der Road, Taichung, Taiwan, Tel: +886 4 22052121, ext. 2008. E-mail: d6781@mail.cmuh.org.tw. Yang-Chang Wu, School of Pharmacy, College of Pharmacy, China Medical University, 91, Hsueh-Shih Road, Taichung, Taiwan, Tel: +886 4 22053366, ext. 7832 E-mail: yachwu@mail.cmu.edu.tw.

Fig. S1: Full-length blots from Fig. 2.

Fig. 2a

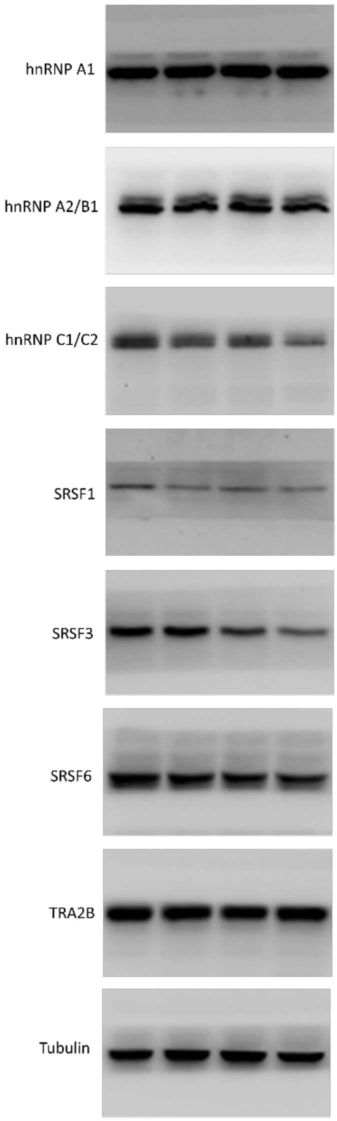

Fig. 2b

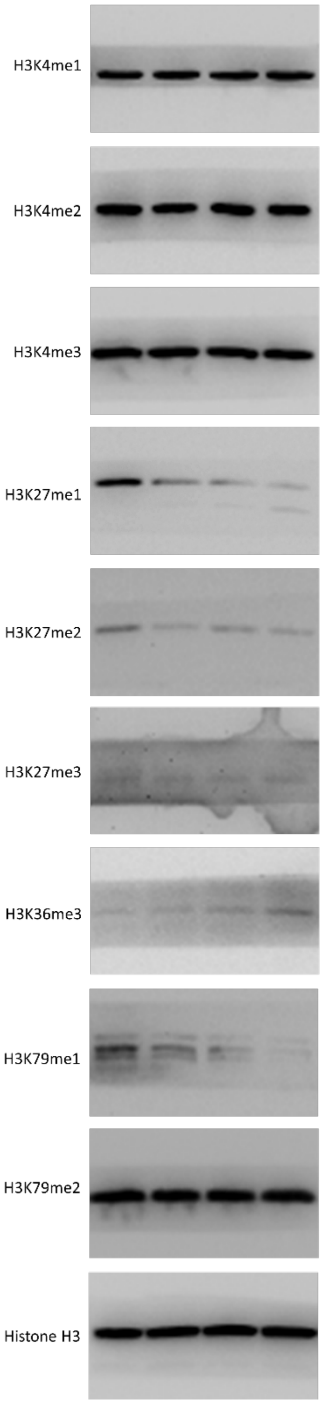

Fig. 2c

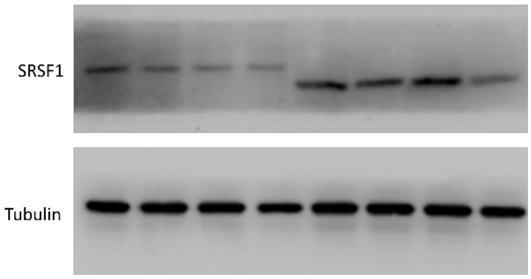

Fig. S2: Full-length blots from Fig. 3.

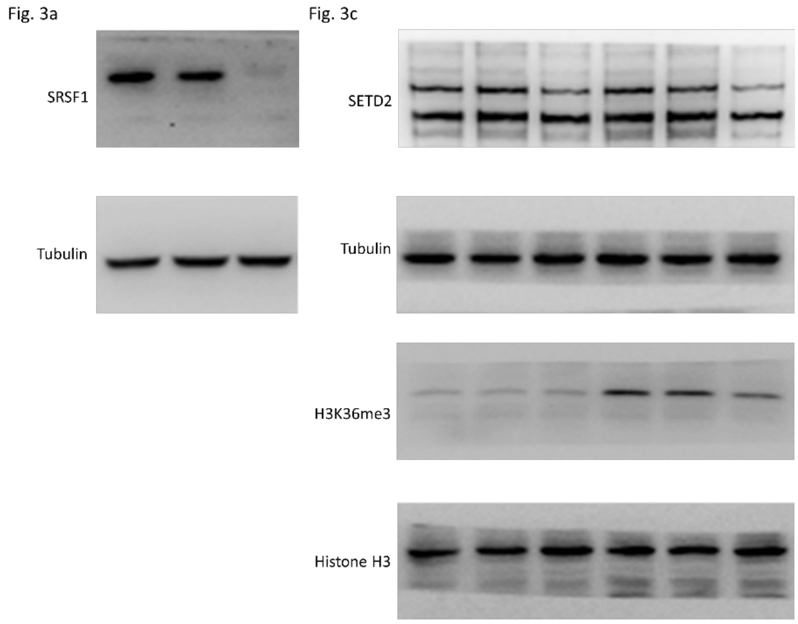

Fig. S3: Full-length blots from Fig. 4.

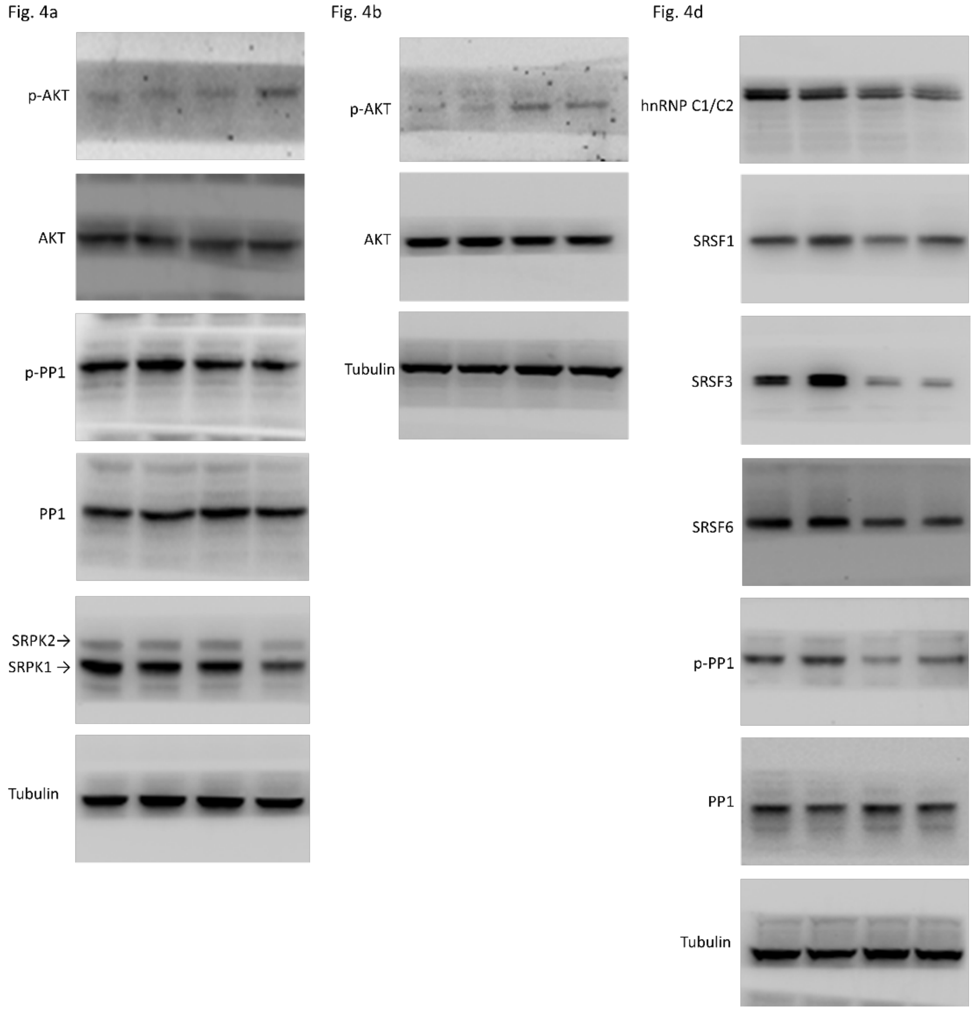

Fig. S4: Full-length blots from Fig. 6.

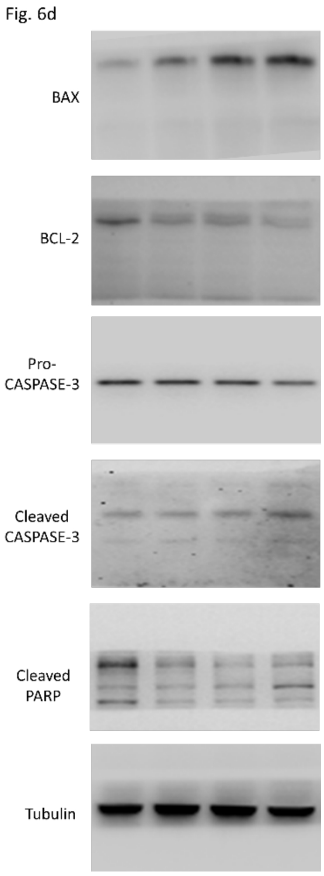

Table S1: Information about the Small interfering RNA used in the study

Gene	Sequences
SRSF1 (sense)	GCCCAGAAGUCCAAGUUAUTT
SRSF1 (antisense)	AUAACUUGGACUUCUGGGCTT
SEDT2 (sense)	GCAGGACACUAUAUCUAAUTT
SEDT2 (antisense)	AUUAGAUAUAGUGUCCUGCTT
Scrambled control (sense)	UUCUCCGAACGUGUCACGUTT
Scrambled control (antisense)	ACGUGACACGUUCGGAGAATT

Table S2: Information about the primers used in RT-PCR

Gene	Forward primer	Reverse primer
APAF1	ATGCGACATCAGCAAATGAG	AATGTCCTCTGCAATCAGCC
CCAR1	AAGAGAAGGATGATGGTGAAGC	CGTTGAAAAGTTCCGCAAAC
HIPK3	AGCCTGCCACTACCAAGAAA	CAGCAATTTCTTGCCTCTCC
RIPK1	ACAGGCCAGATGTGGATGAC	CTGAATTTGACCGGCTTGAA
SMAC/DIABLO	AGTAACCCTGTGTGCGGTTC	CTGCCACACTTCATCTTCCTC
SURVIVIN	CACCGCATCTCTACATTCAA	CACTTTCTCCGCAGTTTCCT
